# MCC950 Reduces the Anxiodepressive-like Behaviors and Memory Deficits Related to Paclitaxel-Induced Peripheral Neuropathy in Mice

**DOI:** 10.3390/antiox14020143

**Published:** 2025-01-25

**Authors:** Ignacio Martínez-Martel, Sylmara Esther Negrini-Ferrari, Olga Pol

**Affiliations:** 1 Grup de Neurofarmacologia Molecular, Institut de Recerca Sant Pau, 08041 Barcelona, Spain; 2 Grup de Neurofarmacologia Molecular, Institut de Neurociències, Universitat Autònoma de Barcelona, 08193 Barcelona, Spain

**Keywords:** anxiety, depression, MCC950, memory, neuropathic pain, NLRP3 inflammasome, oxidative stress, paclitaxel

## Abstract

Chemotherapy-induced peripheral neuropathy and the accompanying affective disorders are serious side effects, and their resolution is not guaranteed. Oxidative stress and elevated levels of Nod-like receptor protein 3 (NLRP3) have been detected in the peripheral and central nervous systems of animals with neuropathic pain provoked by several antineoplastic drugs, such as paclitaxel (PTX). Several studies have further indicated that NLRP3 inflammasome inhibition could be an approach for treating chronic pain, but its impact on the anxiodepressive-like behaviors and memory deficits related to PTX-provoked neuropathy has not yet been investigated. MCC950 is a potent and specific inhibitor of the NLRP3 pathway that acts through inhibiting NLRP3 activation and inflammasome formation. We hypothesized that the administration of MCC950 could alleviate the affective and cognitive disorders accompanying PTX-provoked neuropathy. Using male C57BL/6 mice, we assessed the effects of MCC950 on the mechanical and thermal allodynia, anxiodepressive-like behavior, and memory deficits incited by this taxane. The results indicated that the intraperitoneal administration of 10 mg/kg of MCC950 twice daily for three consecutive days fully reversed the PTX-induced mechanical and thermal allodynia. This treatment also completely attenuated the anxiolytic (*p* < 0.004) and depressive-like behaviors (*p* < 0.022) and memory deficits (novel object recognition test; *p* < 0.0018) incited by PTX. These actions were mainly achieved through blocking NLRP3 inflammasome activation in the sciatic nerve, amygdala, and hippocampus, and oxidative stress in the amygdala and hippocampus. MCC950 also normalized the p-ERK 1/2 overexpression in the sciatic nerve and apoptotic responses in the sciatic nerve and the amygdala. This study suggests that MCC950 might be a promising treatment for PTX-induced mental illnesses and neuropathy.

## 1. Introduction

Paclitaxel (PTX) is an antineoplastic drug that is currently used for the management of lung cancer, breast cancer, ovarian carcinoma, pancreatic malignancies, and melanoma [1]. However, despite its multiple clinical benefits, treatment with this chemotherapy agent is associated with the development of peripheral neuropathy, an undesirable effect for cancer patients that can persist for a long period of time after the completion of the treatment [2,3]. It is also well known that chemotherapy-induced neuropathy is linked to significant psychological problems, such as anxiety, depression, and memory lapses, which can reinforce pain sensitivity, lengthen the duration of chemotherapy, and interfere with proper cure [4]. Unfortunately, the current therapies to reduce the neuropathy and mental illness that come with chemotherapy are limited, posing significant clinical challenges [5].

The Nod-like receptor protein 3 (NLRP3) inflammasome is involved in several diseases, including diabetes, atherosclerosis, Alzheimer’s disease, inflammatory bowel disease, and multiple sclerosis [6,7]. In recent years, the NLRP3 inflammasome has gained considerable importance and is considered a key player in the development of chronic pain [8,9]. Accordingly, several studies have demonstrated its activation in some pain conditions, such as burn pain [10], fibromyalgia [11], osteoarthritis [12], and neuropathic pain caused by nerve injury [13] or linked to diabetes [14]. In addition, patients with gout or rheumatoid arthritis were found to have an activated and up-regulated NLRP3 system, and its inhibition provided pain relief [8].

Moreover, the intrathecal injection of siRNA against the Nlrp3 gene prevented bortezomib-induced mechanical allodynia in mice, and the overexpression of the Nlrp3 gene in the dorsal root ganglia of these animals contributed to the development of mechanical allodynia [15]. However, the possible contribution of the NLRP3 inflammasome to PTX-induced mechanical and thermal allodynia has not yet been evaluated.

It is interesting to note that the NLRP3 inflammasome is also implicated in numerous mental illnesses, including anxiety- and depressive-like behaviors linked to spinal cord injuries [16] or persistent inflammation [17], and in the cognitive deficiencies related to Alzheimer’s [18]. However, its potential role in the psychological and memory deficiencies associated with PTX-induced neuropathic pain remains unexplored.

PTX injections have been shown to induce NLRP3 inflammasome activation in the dorsal root ganglia and the sciatic nerve, and this stimulation is linked to the onset of neuropathic pain [19]. Oxidative stress also plays a crucial role in the development of PTX-induced neuropathy [20]. Previous works have demonstrated the up-regulation of various oxidative stress markers and the down-regulation of the antioxidant enzymes heme oxygenase 1 (HO-1), NAD(P)H quinone dehydrogenase 1 (NQO1), and superoxide dismutase 1 (SOD-1) in animals with chemotherapy-induced neuropathic pain [21]. Oxidative stress also promotes NLRP3 inflammasome activation, and the allodynia triggered by PTX can be reduced by inhibiting reactive oxygen species production [19]. The activation of the endogenous antioxidant pathway can also reverse PTX-induced neuropathy and the accompanying anxiodepressive-like behaviors [22].

The extracellular signal-regulated kinase (ERK 1/2) signaling pathway is also involved in the neuropathy induced by PTX. Previous studies found increased p-ERK 1/2 expression in the peripheral and central nervous systems of animals injected with PTX [22,23,24]. Furthermore, the administration of a selective inhibitor of ERK 1/2 activation (magnolin) reduced the cold allodynia caused by PTX [24]. Other researchers have also found that PTX-induced cognitive impairments may be caused by the changes in synaptic plasticity provoked by this antineoplastic agent [25]. In addition, the multiple alterations provoked by PTX (for example, the inflammatory responses, disturbances to the antioxidant system, and ERK 1/2 phosphorylation) activate apoptosis in the central and peripheral nervous systems [26,27]. Furthermore, increased levels of BAX in the dorsal root ganglia [22] and necroptosis in the hippocampus have been found in animals given PTX [25].

MCC950, a selective inhibitor of the NLRP3 inflammasome, has shown promising therapeutic effects in several preclinical models through its ability to reduce the inflammatory responses induced by proinflammatory cytokines such as IL-1β and IL-18 [9,28,29]. Repeated treatment with MCC950 attenuated cancer-induced bone-pain-related mechanical allodynia by reversing NLRP3 inflammasome up-regulation in the spinal cord [30]. The administration of MCC950 also attenuated the mechanical allodynia in a mouse model of experimental autoimmune prostatitis [31] and in animals with vincristine-evoked neuropathic pain [32]. However, the effects of MCC950 on the allodynia and emotional and memory deficits incited by PTX have not been evaluated.

Recent studies have revealed analgesic effects of molecular hydrogen in different pain models, especially in neuropathic pain induced by nerve injury or chemotherapy [23,33]. The administration of hydrogen-rich water (HRW), in addition to producing potent antioxidant effects, also induced notable anti-inflammatory effects by inhibiting the expression of the NLRP3 inflammasome and several proinflammatory mediators, such as TNF-α, IL-1β, and IL-6, in animals with inflammatory and neuropathic pain due to PTX administration [34,35]. Due to the positive effects demonstrated by HRW, the possible potentiation of the analgesic actions of MCC950 through its co-administration with HRW was also investigated in this study.

In summary, several studies have evaluated the analgesic actions of MCC950 in different animal pain models [30,31,32], but the effect of this NLRP3 inflammasome inhibitor on the affective and cognitive disorders accompanying PTX-provoked neuropathic pain and its main mechanisms of action have not been investigated. In addition, it is well known that molecular hydrogen interacts with other molecules, such as the antioxidant enzyme HO-1, potentiating its painkiller properties [33]; however, the interaction between MCC950 and HRW has not been evaluated. We hypothesized that treatment with MCC950 might inhibit the neuropathic and mental deficits incited by PTX through regulating the endogenous inflammatory and antioxidant systems, and, moreover, that the co-administration of HRW would potentiate the analgesic actions of MCC950 under these experimental conditions.

Using male C57BL/6 mice injected with PTX, we assessed the effects of MCC950 on (1) the allodynia (mechanical and thermal), anxiety- and depressive-like behaviors, and memory deficits incited by this antineoplastic agent and (2) the inflammatory and oxidative responses, as well as the plasticity changes and apoptosis caused by this taxane in the sciatic nerve, amygdala, and hippocampus. (3) We also evaluated the antiallodynic effects induced by the combination of MCC950 and HRW.

## 2. Materials and Methods

### 2.1. Animals

Male C57BL/6 mice (5–6 weeks old) from Envigo Laboratories (Barcelona, Spain) were used in this study. They were housed at 22 ± 1 °C with a relative humidity of 66% and a 12 h light/dark cycle and were provided with food and water ad libitum. The animals (136 in total) were housed in polypropylene cages containing an enriched environment, including a carton hut and cellulose fragments.

This study was approved by the local Committee for Animal Use and Care of the Autonomous University of Barcelona (ethical approval code: 4581), and all procedures were conducted in accordance with the guidelines of the European Commission’s directive 2010/63/EC and the Spanish Law (RD 53/2013) regulating animal research. All efforts were made to minimize both the quantity and the suffering of the animals.

### 2.2. Experimental Procedures

The contribution of NLRP3 to PTX-provoked neuropathy and affective and cognitive disorders was evaluated by assessing the effects of MCC950 on the mechanical and thermal allodynia, anxiodepressive-like behaviors, and memory loss in animals given PTX.

**Figure 1:**
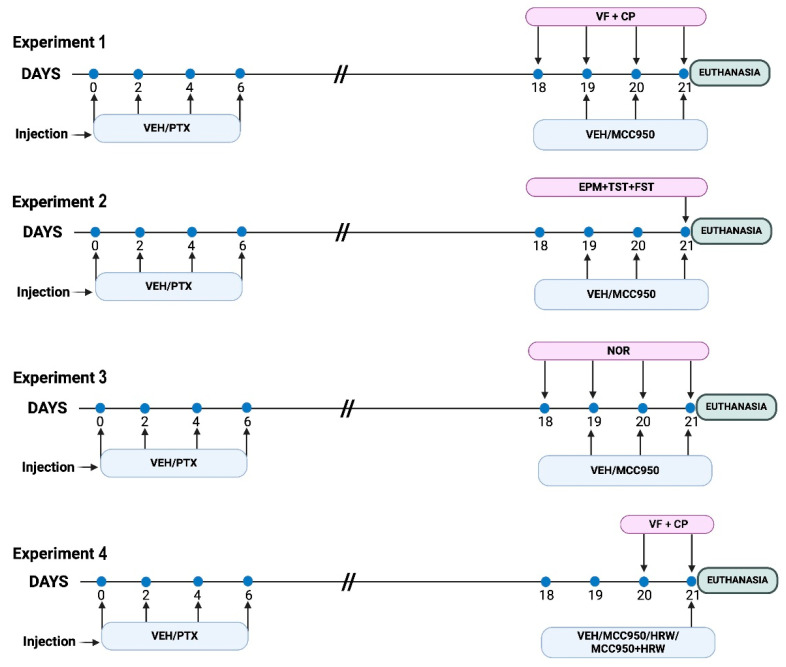
Schematic representation of the experimental procedures. VEH: vehicle; PTX: paclitaxel; VF: von Frey filament test; CP: cold plate test; EPM: elevated plus maze; TST: tail suspension test; FST: forced swimming test; NOR: new object recognition test.

For this purpose, four experimental groups of animals were injected with vehicle (VEH) or PTX (Figure 1). In the first experiment, VEH- and PTX-injected mice were intraperitoneally treated with 10 mg/kg of MCC950 or VEH twice daily for 3 consecutive days at 19, 20, and 21 days. Mechanical and thermal allodynia were assessed 18, 19, 20, and 21 days after the first injection of PTX or VEH and 1 h after the administration of MCC950 or VEH [12] (n = 6 animals per group). At the end of the experiments, the animals were euthanized and the effects of MCC950 on the expression of the NLRP3 inflammasome, p-ERK-1/2, oxidative and antioxidant proteins, and the apoptosis indicator BAX in the sciatic nerves, amygdalae, and hippocampi of these animals were examined using Western blots (n = 6 samples per group).

In the second experiment, in PTX- and VEH-injected mice that received the same doses and pattern of administration of MCC950 or VEH as those in experiment 1, we evaluated the effects of the NLRP3 inflammasome inhibitor on the anxiety- and depressive-like behaviors caused by PTX using the elevated plus maze (EPM) test and using the tail suspension test (TST) and the forced swimming test (FST), respectively. The tests were performed on day 21 (n = 8 animals per group).

In the third experiment, in PTX- and VEH-injected mice that received the same doses and pattern of administration of MCC950 or VEH that were utilized in experiment 1, the effect of the NLRP3 inflammasome inhibitor on the memory deficits provoked by PTX was assessed using the novel object recognition (NOR) test, which was performed on day 21 (n = 8 animals per group).

In the fourth experiment, to study whether HRW treatment could increase the antiallodynic effects of a single dose of MCC950, the effects produced by the acute co-administration of MCC950 (10 mg/kg) and HRW (0.15 mM) on the mechanical and cold allodynia provoked by PTX were assessed on day 21. Von Frey filament and cold plate tests were conducted 1 h after the MCC950 or HRW injection, given alone or in combination, following the protocol from a previous study [34] (n = 6 animals per group).

### 2.3. PTX Injection

The mice were intraperitoneally administered a 2 mg/kg dose of PTX (Tocris Bioscience, Bristol, UK) diluted in a mixture of Cremophor EL (Sigma-Aldrich, St. Louis, MO, USA), ethanol, and saline (SS, 0.9% NaCl) in a ratio of 1:1:18; the mice were injected four times every other day (on days 0, 2, 4, and 6), in accordance with the study protocol of Toma et al. (2017) [36]. The control animals were given the same volume of vehicle solution using the same protocol.

### 2.4. Drugs

MCC950 (Sigma-Aldrich, St. Louis, MO, USA) was dissolved in 0.9% NaCl. The HRW was prepared using a hydrogen water generator from Hydrogen (Osmo-star Soriano S.L., Alicante, Spain). Both compounds were prepared before use and intraperitoneally administered in a volume corresponding to 10 mL/kg. The controls received an equal volume of VEH.

### 2.5. Allodynia Tests

Mechanical hyperalgesia was quantified by measuring the hind-paw withdrawal response to von Frey filament (North Coast Medical, Inc., San Jose, CA, USA) stimulation and applying the up–down paradigm [37]. The mice were placed in separate methacrylate cylinders (20 cm high × 9 cm wide) on an elevated wire platform through which filaments were applied to both hind paws. A 0.4 g filament was used first, and the strength of the subsequent filament was decreased if the animal responded or increased if the animal did not respond. The threshold of response was estimated using an Excel program which included curve fitting of the data.

Cold allodynia was assessed utilizing a cold plate analgesiometer (Ugo Basile, Gemonio, Italy) at 4 ± 0.5 °C, and the number of both hind-paw elevations was recorded for 5 min.

### 2.6. Emotional Behavior Tests

The evaluation of anxiety-like behaviors was conducted using an EPM apparatus, which consisted of four arms measuring 5 × 35 cm that were elevated at a height of 45 cm. Two arms were open and two were enclosed by 15 cm high walls. Each mouse was placed in the central neutral area facing one of the open arms and allowed to explore the maze for 5 min. The sessions were recorded using a digital camera, and the total number of visits to the open and closed arms and the percentage of time spent in the open arms were measured [38].

In accordance with previous investigations, the TST and FST were used to assess depressive-like behaviors [39,40]. In the TST, the animals were suspended at a height of 35 cm from the ground by attaching adhesive to the tips of their tails. We used a digital camera to record their movements, and the time spent immobile was measured for 6 min. In the FST, each mouse was placed in a Plexiglas cylinder (25 cm high and 10 cm wide) containing water at a depth of 10 cm at 24 °C. The activity of the animal was recorded for 6 min, and the time that it spent immobile during the last 4 min was determined.

### 2.7. Cognitive Behavior Test

The assessment of novel object recognition memory was conducted using a gray box (44 × 44 cm) consisting of four walls and a non-reflective base [41]. This test was conducted in four sessions, which lasted 10 min each. The animals were habituated to the empty box for two days. On day 3, the mice were placed in the box again and two identical objects were shown. On day 4, the mice were reintroduced into the box and one of the familiar objects was replaced by a novel one. The time spent exploring both the new and familiar objects was quantified. Cognitive behavior was measured using the discrimination index, which was calculated using the following formula: (time exploring novel object − time exploring familiar object)/(time exploring novel plus familiar object) × 100. A high discrimination index indicates good recognition memory.

In these experiments, before starting the tests, the mice were habituated to the testing room for 1 h, and the equipment was carefully cleaned between subjects. The experiments were carried out by investigators who were blind to the experimental conditions.

### 2.8. Western Blot Analysis

Twenty-one days after the PTX or VEH injection, the animals were euthanized by cervical dislocation, and the sciatic nerve, amygdala, and hippocampus were dissected and stored at −80 °C until further use. The tissues were homogenized using sonication in RIPA Buffer (Sigma-Aldrich, St. Louis, MO, USA), solubilized (1 h at 4 °C), sonicated (10 s), and centrifuged (700 g for 20 min at 4 °C). Equal amounts of protein (60 μg) were loaded into 4% stacking/12% separating sodium dodecyl sulfate–polyacrylamide gels and electrophoretically transferred onto polyvinylidene fluoride membranes. The membranes were blocked for 75 min in blocking buffer (phosphate-buffered saline (PBS) + 5% non-fat dry milk, PBS + Tween 20 + 5% bovine serum albumin (BSA), or Tris-buffered saline + Tween 20 + 5% non-fat dry milk or BSA) and incubated overnight at 4 °C with specific primary antibodies (Table 1). After washing, the membranes were incubated for 1 h at room temperature with the secondary antibody (GE Healthcare, Little Chalfont, UK). Then, the membranes were developed using ECL kit reagents (GE Healthcare, Little Chalfont, UK), detected using a Chemidoc MP system (Bio-Rad, Hercules, CA, USA), and analyzed using Image-J software (version 1.8.0; National Institutes of Health, Bethesda, MD, USA).

### 2.9. Statistical Analyses

The SPSS (version 28, IBM, Madrid, Spain) and Prism 8.0 (Graphpad, La Jolla, CA, USA) programs were used for the statistical analyses. The data are displayed as the means ± standard errors of the means (SEMs). The results were analyzed using Bartlett’s test to assess the variance homogeneity and the Shapiro–Wilk test to determine the distribution of the data. The effect of MCC950 on the mechanical and thermal allodynia caused by PTX was evaluated using the three-way repeated-measures ANOVA, with injection, treatment, and time as the variation factors. Since the data were not normally distributed or the variances differed across the groups, the Kruskal–Wallis test followed by Dunn’s post hoc multiple-comparisons test were used to assess the effect of MCC950 on the mechanical and thermal allodynia and the emotional and memory deficits induced by PTX. The differences in the protein levels between the groups were also analyzed using the Kruskal–Wallis test, followed by Dunn’s multiple-comparisons test. The analysis of the antiallodynic effects produced by co-administration of HRW plus MCC950 were performed using a two-way ANOVA, with injection and treatment as the variation factors, followed by the Kruskal–Wallis test and Dunn’s multiple-comparisons test. *p* values < 0.05 were considered statistically significant.

## 3. Results

### 3.1. The Effects of MCC950 on the Mechanical and Thermal Allodynia Caused by PTX

We evaluated if the activation of the NLRP3 inflammasome contributes to PTX-induced neuropathic pain by assessing the effects of intraperitoneal administration of an NLRP3 inhibitor, MCC950, on PTX-induced mechanical and thermal allodynia using von Frey filament and cold plate tests, respectively.

**Figure 2:**
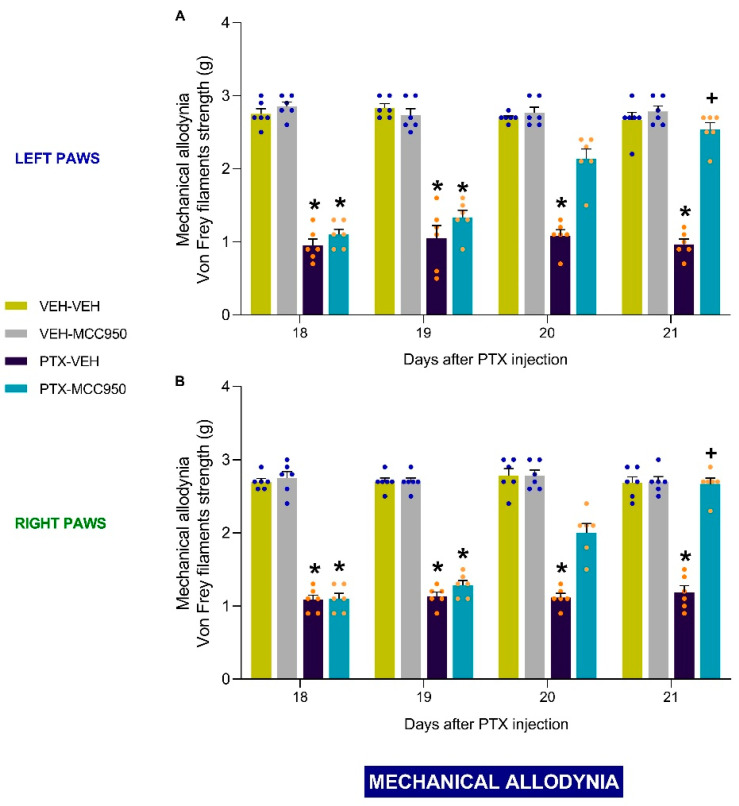
Reversion of the mechanical allodynia triggered by PTX after administration of 10 mg/kg of MCC950 (given intraperitoneally, twice a day on three consecutive days). The data are shown as the von Frey filaments strength (g) exerted on the left (**A**) and right (**B**) paws. In all graphs, the symbols indicate significant differences compared to VEH-VEH or VEH-MCC950 (*) and PTX-VEH (+) mice (*p* < 0.05, Kruskal–Wallis test followed by Dunn’s multiple-comparisons test). The values are the means ± SEMs from 6 animals per group.

**Figure 3:**
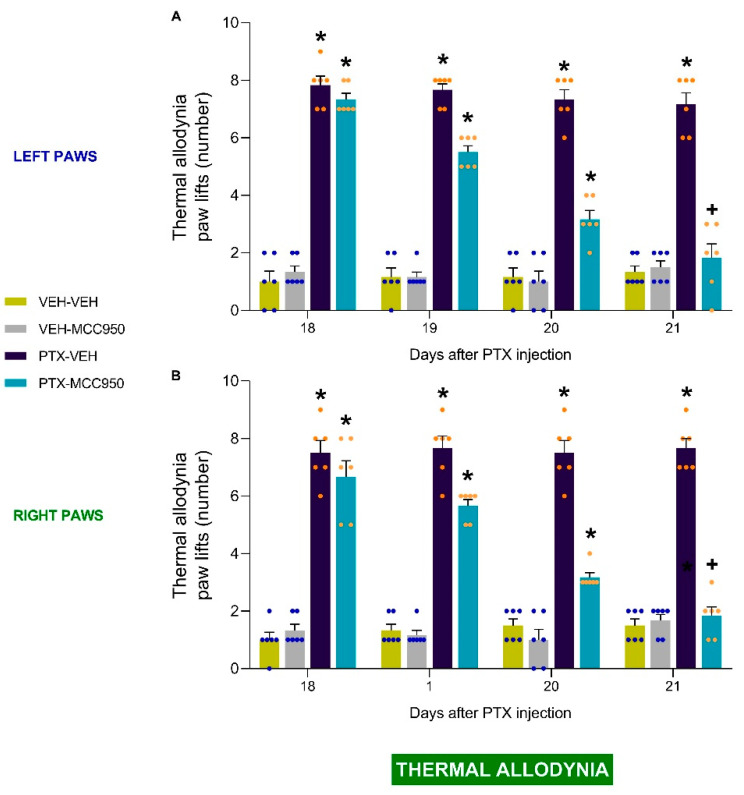
Reversion of the thermal allodynia triggered by PTX after administration of 10 mg/kg of MCC950 (given intraperitoneally twice a day on three consecutive days). The data are shown as the numbers of paw lifts for the left (**A**) and right (**B**) paws. In all graphs, the symbols indicate significant differences compared to VEH-VEH or VEH-MCC950 (*) and PTX-VEH (+) mice (*p* < 0.05, Kruskal–Wallis test followed by Dunn’s multiple-comparisons test). The values are the means ± SEMs of 6 animals per group.

In both tests, the three-way repeated-measures ANOVA revealed significant effects of injection, treatment, time, and the interactions between them (*p* < 0.0001). Our results indicated that the administration of MCC950 reversed the low threshold for paw withdrawal in response to von Frey filaments (Figure 2A,B; vs. PTX-injected animals receiving VEH: *p* < 0.0001) and the increased number of paw lifts in the cold plate test (Figure 3A,B; vs. PTX-injected animals receiving VEH: *p* < 0.0001) in the left and right hind paws compared to mice injected with PTX alone. In both paws, the complete reversal of the mechanical and thermal allodynia was observed after 3 days of treatment. The administration of MCC950 did have any effect on the paws of VEH-injected animals (Figure 2A,B and Figure 3A,B).

### 3.2. The Effects of MCC950 on the Anxiodepressive-like Behaviors and Memory Loss Linked to PTX

Our results confirmed that at three weeks after the PTX injection, the mice displayed anxious- and depressive-like behaviors and memory deficits. Therefore, we evaluated if inhibition of NLRP3 inflammasome activation could reduce these affective and cognitive disorders.

**Figure 4:**
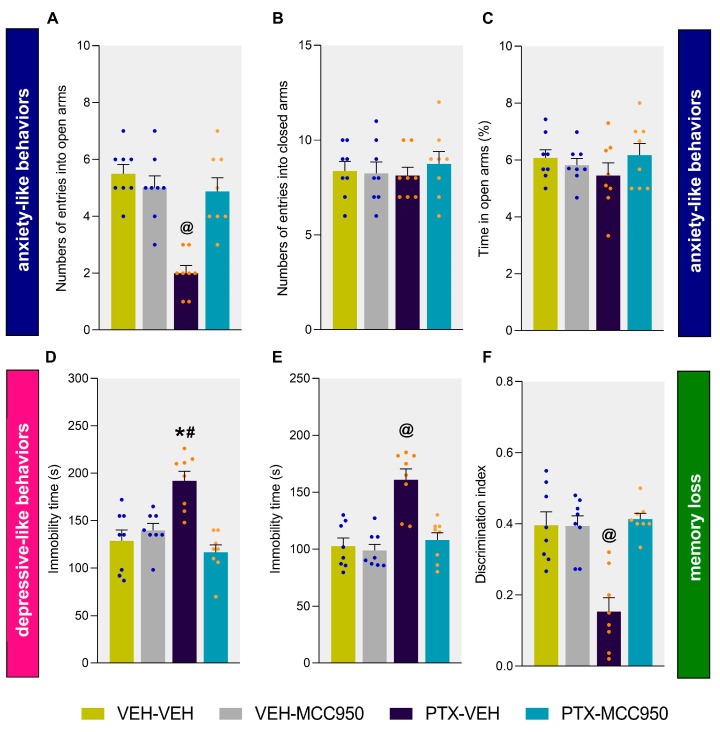
Inhibition of the anxiodepressive-like behaviors and memory loss associated with PTX after the intraperitoneal administration of 10 mg/kg of MCC950 on 3 consecutive days. The number of entries into the open (**A**) and closed arms (**B**) and the proportion of time spent in the open arms (**C**) in the EPM. The time that the animals remained immobile (s) in the TST (**D**) and FST (**E**) and the discrimination index in the object recognition test (**F**). In all graphs, the symbols indicate significant differences compared to VEH-VEH (*), PTX-MCC950 (#), and the other groups (@) (*p* < 0.05, Kruskal–Wallis test followed by Dunn’s multiple-comparisons test). The data are expressed as the mean values ± SEMs of 8 animals for each group.

The EPM test was used to assess the anxiety-like behaviors of the animals; the results showed a reduced number of entries into the open arms by the mice injected with PTX (Figure 4A; vs. mice given VEH-VEH: *p* < 0.004); this was reversed when the mice were co-treated with MCC950. These results reveal an anxiolytic action of this compound and the important role played by the NLRP3 inflammasome in the development of anxiety-like behaviors associated with PTX chemotherapy. There were no differences between the groups regarding the number of entries to the closed arms (Figure 4B) or the percentage of time spent in the open arms (Figure 4C).

We also investigated whether the MCC950 treatment could reverse the depressive-like behavior provoked by PTX on day 21 after the PTX injection. Our data showed that there was an increase in the immobile time of PTX-injected animals in the TST (Figure 4D; vs. VEH-VEH-treated mice: *p* < 0.0009) and FST (Figure 4E; vs. VEH-VEH-treated mice: *p* < 0.0022), which was completely normalized by giving MCC950 on three consecutive days. These outcomes revealed the anxiolytic and antidepressant effects of MCC950 in PTX-injected animals.

PTX-induced neuropathy is commonly accompanied by memory dysfunction. Our findings confirmed that memory deficits were induced by the PTX treatment by demonstrating a reduction in the discrimination index in PTX-injected subjects in the object recognition test (Figure 4F; vs. VEH-VEH-treated mice: *p* < 0.0018). Remarkably, this decrease in the discrimination index was reversed with the administration of MCC950, thus showing the rescue of the memory deficits by this drug in mice given PTX.

### 3.3. The Effects of MCC950 on the Inflammatory, Oxidative, Plasticity, and Apoptotic Changes Provoked by PTX in the Sciatic Nerve, Amygdala, and Hippocampus

Considering the crucial role of inflammation and oxidative stress in the development of neuropathy after PTX chemotherapy, we evaluated the impact of the pharmacological inactivation of the NLRP3 inflammasome on the expression of markers of oxidative stress, synaptic plasticity, and apoptosis.

**Figure 5:**
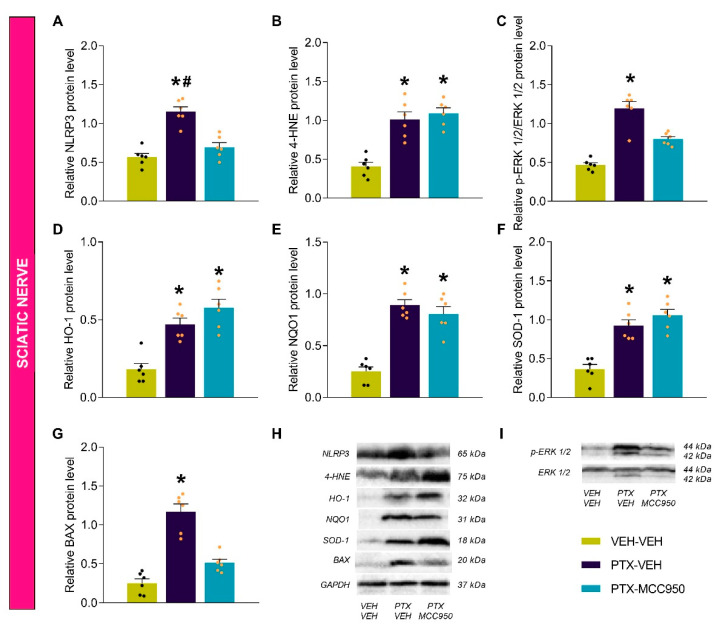
Effects of MCC950 treatment on the protein levels of NLRP3 (**A**), 4-HNE (**B**), p-ERK 1/2 (**C**), HO-1 (**D**), NQO1 (**E**), SOD-1 (**F**), and BAX (**G**) in the sciatic nerves of PTX-injected mice. VEH-injected mice given VEH were used as controls. (**H**,**I**) Representative blots of these proteins. The symbols indicate significant differences vs. VEH-VEH (*) and PTX-MCC950 (#) (*p* < 0.05; Kruskal–Wallis test followed by Dunn’s multiple-comparisons test). The results are shown as the means ± SEMs of 6 samples/group.

**Figure 6:**
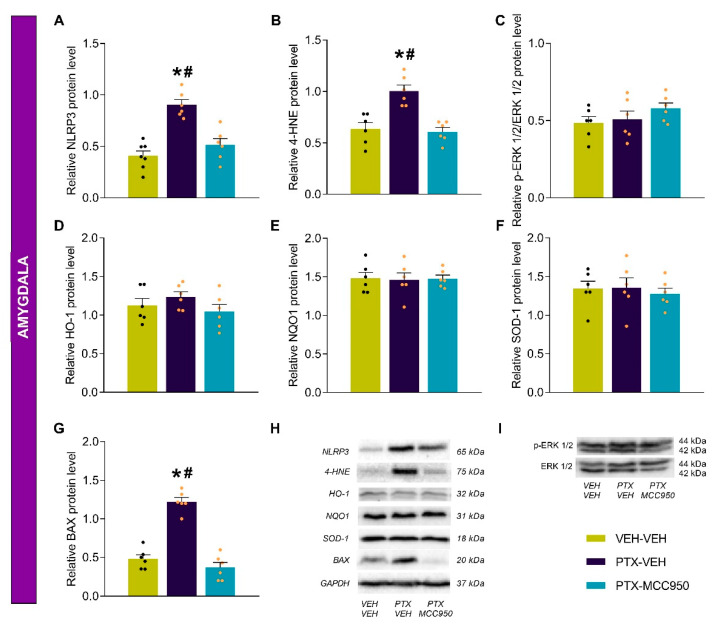
Effects of MCC950 treatment on the protein levels of NLRP3 (**A**), 4-HNE (**B**), p-ERK 1/2 (**C**), HO-1 (**D**), NQO1 (**E**), SOD-1 (**F**), and BAX (**G**) in the amygdalae of PTX-injected mice. VEH-injected mice given VEH were used as controls. (**H**,**I**) Representative blots of these proteins. The symbols represent significant differences vs. VEH-VEH (*) and PTX-MCC950 (#) (*p* < 0.05; Kruskal–Wallis test followed by Dunn’s multiple-comparisons test). The results are shown as the means ± SEMs of 6 samples/group.

**Figure 7:**
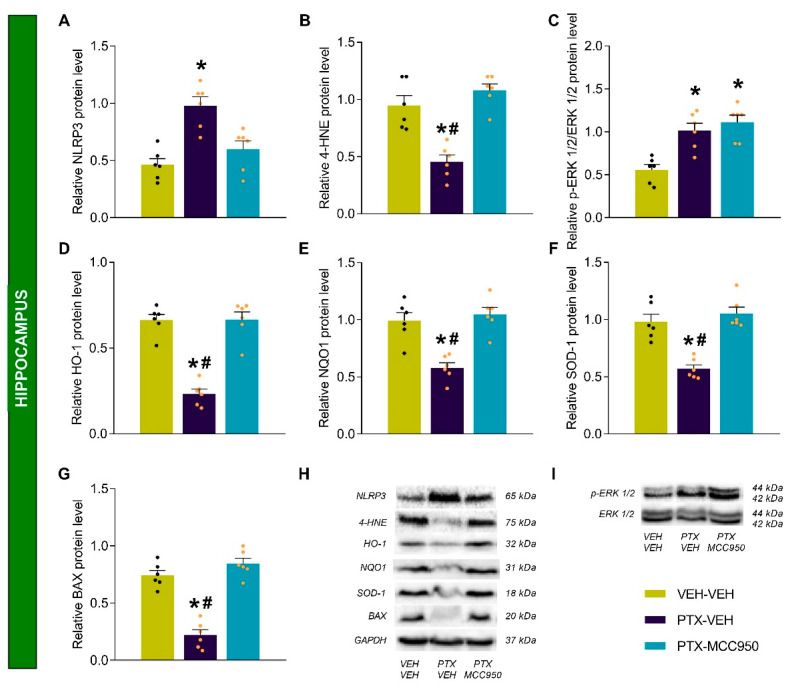
Effects of MCC950 treatment on the protein levels of NLRP3 (**A**), 4-HNE (**B**), p-ERK ½ (**C**), HO-1 (**D**), NQO1 (**E**), SOD-1 (**F**), and BAX (**G**) in the hippocampi of PTX-injected mice. VEH-VEH-injected mice were used as controls. (**H**,**I**) Representative blots of these proteins. The symbols indicate significant differences vs. VEH-VEH (*) and PTX-MCC950 (#) mice (*p* < 0.05; Kruskal–Wallis test followed by Dunn’s multiple-comparisons test). The results are shown as the means ± SEMs of 6 samples/group.

As expected, higher levels of the NLRP3 inflammasome were detected in the sciatic nerves (Figure 5A; *p* < 0.0001), amygdalae (Figure 6A; *p* < 0.0002), and hippocampi (Figure 7A; *p* < 0.0002) of PTX-injected mice compared to VEH-VEH-treated mice. These levels were normalized by the administration of MCC950. The oxidative stress induced by PTX, demonstrated by the increased levels of 4-HNE (an oxidative stress marker) in the sciatic nerve (Figure 5B; vs. VEH-VEH-treated mice: *p* < 0.0005) and amygdala (Figure 6B; vs. VEH-VEH-treated mice: *p* < 0.0005), was attenuated by the MCC950 treatment in the amygdala but not in the sciatic nerve. In contrast, down-regulation of 4-HNE was detected in the hippocampi of PTX-injected animals (Figure 7B; vs. VEH-VEH-treated mice: *p* < 0.0003), which was normalized by the administration of MCC950.

Several studies have demonstrated increased ERK 1/2 phosphorylation in animals that received chemotherapy with PTX. Our results also detected increased p-ERK 1/2 levels in the sciatic nerves (Figure 5C; *p* < 0.001) and hippocampi (Figure 7C; *p* < 0.0006) of PTX-injected mice compared with those treated with VEH-VEH, but not in the amygdalae (Figure 6C). The high levels of p-ERK 1/2 were only reversed by the MCC950 treatment in the sciatic nerve.

Given that there are interactions between inflammation and oxidative stress during chronic pain, the effects of the NLRP3 inflammasome inhibitor MCC905 on the antioxidant system were also assessed. Our data showed that, compared to VEH-VEH-treated mice, treatment with MCC905 maintained the high levels of HO-1 (Figure 5D; *p* < 0.0001), NQO1 (Figure 5E; *p* < 0.0004), and SOD-1 (Figure 5F; *p* < 0.0002) induced by PTX in the sciatic nerve; it did not alter the expression of these enzymes in the amygdala (Figure 6D–F), but it did normalize the down-regulation of HO-1 (Figure 7D; *p* < 0.0005), NQO1 (Figure 7E; *p* < 0.0004), and SOD-1 (Figure 7F; *p* < 0.0002) induced by PTX in the hippocampus.

Finally, we assessed the effects of MCC950 on the apoptotic responses generated by PTX through analyzing the protein levels of BAX. While the expression of BAX increased in the sciatic nerves (Figure 5G; *p* < 0.0001) and amygdalae (Figure 6G; *p* < 0.0002) of PTX-injected mice compared to VEH-VEH-treated mice, its expression decreased in the hippocampi of these animals (Figure 7G; *p* < 0.0002). Treatment with MCC950 normalized the up-regulation of BAX in the sciatic nerve and amygdala and the down-regulation of BAX in the hippocampus.

### 3.4. The Effects of HRW on the Antiallodynic Actions of MCC950 in PTX-Injected Mice

**Figure 8:**
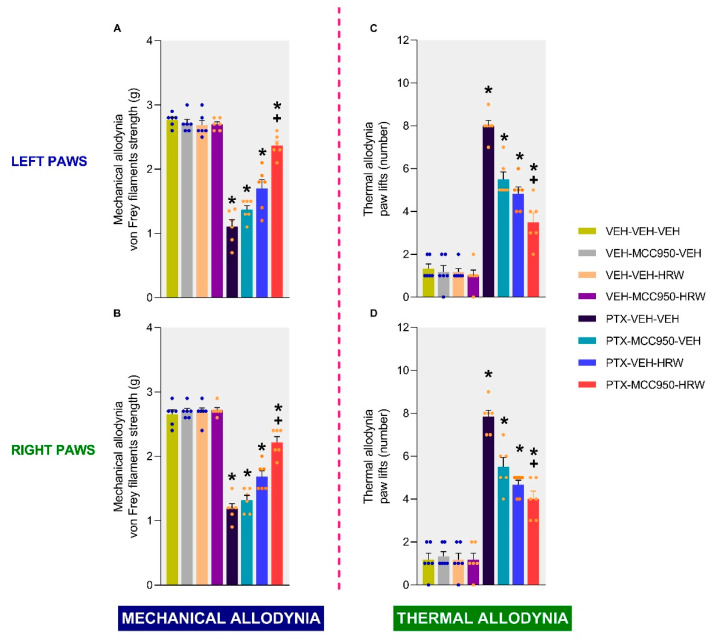
Effects of the acute intraperitoneal administration of MCC950 (10 mg/kg) combined with HRW (0.15 mM) on the mechanical and thermal allodynia on day 21 after PTX injection. The data are presented as the von Frey filament strength (g) on the left (**A**) and right (**B**) paws and the number of paw lifts for the left (**C**) and right (**D**) paws. In all graphs, the symbols indicate significant differences compared to the respective VEH- (*) and PTX-VEH-VEH-treated mice (+) (*p* < 0.05, Kruskal–Wallis test followed by Dunn’s multiple-comparisons test). The results are shown as the means ± SEMs of 6 animals per group.

At 21 days after PTX injection, we further evaluated if the acute administration of HRW (0.15 mM) could enhance the antiallodynic actions induced by a single administration of 10 mg/kg of MCC950 (Figure 8).

For each test and paw analyzed, the two-way ANOVA revealed significant effects of injection (*p* < 0.0010), treatment *p* < 0.0010), and the interactions between them (*p* < 0.0010). Our results revealed that the co-treatment of HRW with MCC950 enhanced the inhibition of the mechanical allodynia induced by each treatment alone, both in the left (Figure 8A; *p* < 0.0001) and right hind paws (Figure 8B; *p* < 0.0001). Moreover, the thermal antiallodynic effects produced by the acute administration of MCC950 were also stronger in the left (Figure 8C; *p* < 0.0001) and right hind paws (Figure 8D; *p* < 0.0001) of animals co-treated with HRW. In both tests and paws, MCC950 and HRW, given together or individually, failed to produce any effect in the VEH-injected animals.

## 4. Discussion

Neuropathy resulting from chemotherapy with PTX causes significant distress to cancer patients, and there is no effective treatment for this type of neuropathy or its associated mental disorders. This study revealed that the pharmacological inhibition of the NLRP3 inflammasome not only attenuated the mechanical and thermal allodynia caused by PTX but also the anxiety- and depressive-like behaviors and memory deficits associated with PTX-induced neuropathy. These effects were mainly produced by inhibiting the up-regulation of the NLRP3 inflammasome in the sciatic nerve, amygdala, and hippocampus and the oxidative stress marker 4-HNE in the amygdala. This treatment also maintained high levels of the antioxidant proteins HO-1, NQO1, and SOD-1 in the sciatic nerve and normalized their down-regulation in the hippocampus. The administration of MCC950 also inhibited ERK 1/2 phosphorylation in the sciatic nerve and the apoptotic responses caused by PTX in the sciatic nerve and amygdala.

These findings demonstrate the effectiveness of MCC950 in attenuating the allodynia and affective and memory deficits evoked by PTX and reveal the contribution of the NLRP3 inflammasome in the development of PTX-induced neuropathy and its associated adverse effects. Additionally, treatment with HRW increased the antiallodynic actions of MCC950 in PTX-induced neuropathic pain in mice.

The NLRP3 inflammasome is implicated in the pathogenesis of many nociceptive disorders, such as arthritis and neuropathic and inflammatory pain, as well as cancer-induced bone pain [9,30,42]. However, only a few studies have evaluated the contribution of NLRP3 to the neuropathy caused by chemotherapy. Two studies revealed that the administration of the NLRP3 inhibitor MCC950 can attenuate the allodynia caused by oxaliplatin [29] and vincristine in rodents [32]. Our results are in agreement with these findings, with our data demonstrating that MCC950 also attenuated the mechanical and thermal allodynia that manifested in PTX-treated animals. The low threshold of paw withdrawal to von Frey filaments and the increased number of paw lifts on day 18 after the PTX injection were rescued by the repeated administration of MCC950 from day 19 to 21 after the PTX injection. Liu et al. (2018) [15] also demonstrated the prevention of bortezomib-induced mechanical allodynia through the intrathecal injection of siRNA against the Nlrp3 gene and the mechanical allodynia caused by Nlrp3 overexpression in the dorsal root ganglia of these animals [15]. Moreover, the mechanical allodynia provoked by oxaliplatin was also less severe in NLRP3-deficient mice [29].

Our results also showed elevated levels of NLRP3 in the sciatic nerves of PTX-injected animals; this up-regulation was completely inhibited by the repeated administration of MCC950. Interestingly, increased expression of the oxidative marker 4-HNE was also found in the sciatic nerve, supporting the idea that oxidative stress activates the inflammasome pathway, as was also demonstrated in the DRG of animals with osteoarthritis pain [12]. Nonetheless, although MCC950 failed to reverse the increased expression of 4-HNE, this treatment maintained the up-regulation of the antioxidant enzymes HO-1, NQO1, and SOD-1 in the sciatic nerve. Since one of mechanisms underlying chemotherapy-induced neuropathy involves oxidative stress and the induction of antioxidant enzymes, such as HO-1, which can attenuate the mechanical and thermal allodynia caused by PTX [22], the activation of the endogenous antioxidant system by MCC950 suggests that this system could contribute to the antiallodynic effects of this NLRP3 inhibitor.

We also evaluated the effects of MCC950 on the expression of p-ERK 1/2, which is strongly implicated in the development of neuropathic pain [24]. High levels of p-ERK 1/2 were detected in the dorsal root ganglia and spinal cords of animals given oxaliplatin or paclitaxel, and the inhibition of ERK reversed the neuropathic pain induced by both chemotherapeutic agents [43,44]. Accordingly, our results further demonstrated an up-regulation of p-ERK 1/2 in the sciatic nerves of PTX-injected animals and revealed that the administration of MCC950 inhibited ERK 1/2 phosphorylation. Consistent with the findings of previous studies [22], apoptotic responses induced by PTX were observed in the sciatic nerves of animals given this antineoplastic drug, as revealed by the up-regulation of BAX, which was inhibited by the administration of MCC950. These results showed the positive actions of MCC950 in attenuating the plasticity changes and apoptotic responses caused by PTX in the peripheral nervous system. These outcomes also suggest that the NLRP3 inflammasome might be involved in the plasticity changes and apoptotic responses induced by PTX in mice. Moreover, considering the inhibition of neuropathic pain induced by ERK 1/2 inhibitors [43], we postulated that the normalization of p-ERK 1/2 levels by MCC950 might contribute to the effectiveness of this drug in modulating PTX-induced neuropathy in mice.

It is well known that, in addition to peripheral sensitivity, conventional chemotherapy is also associated with notable adverse effects, including anxiety, depression, and memory impairment [4]. However, despite the high prevalence of these symptoms in patients, most preclinical studies have only evaluated the effects of chemotherapy on the allodynia and not on the anxiodepressive-like behaviors and memory deficits, which are critical psychological aspects. In this study, considering that NLRP3 inflammasome activation mediates chronic, unpredictable, mild stress-induced depression [45] and depressive-like behaviors in diabetes patients [46], we evaluated the impact of repeated administration of MCC950 on PTX-associated anxiety- and depressive-like behaviors. Our results demonstrated the ability of MCC950 to normalize the number of entries into the open arms of the EPM, in addition to reducing the time that PTX-injected animals remained immobile in the TST and FST. These data indicate that the treatment with MCC950 also inhibited the anxiodepressive-like behaviors associated with PTX-induced neuropathy, revealing the anxiolytic and antidepressant properties of this treatment.

Our outcomes further proved that the MCC950 treatment normalized the up-regulation of NLRP3 in the amygdalae of mice given PTX. The amygdala is implicated in the control of emotive responses [47], suggesting that the anti-inflammatory effects of MCC950 in this brain area could be responsible for its anxiolytic and antidepressant actions. Other works have also shown that treatments such as sodium butyrate and ultramicronized palmitoylethanolamide can reduce PTX-induced depressive- and anxiety-like behaviors through inhibiting the expression of several NLRP3-derived proinflammatory cytokines in the brain [48,49]. The administration of activators of the antioxidant signaling pathway, such as dimethyl fumarate, 1m, 1b, and 1a, also inhibited the emotional impairments related to neuropathic pain caused by nerve damage through reducing the up-regulation of NLRP3 in the amygdala [50].

Interestingly, the treatment with MCC950 also normalized the increased expression of 4-HNE induced by PTX in the amygdala, showing that the inhibition of NLRP3 inflammasome activation blocked not only the inflammatory responses but also the oxidative stress induced by this chemotherapeutic agent. Thus, MCC950, in addition to its anti-inflammatory actions, also produces antioxidative effects in this brain area. The involvement of oxidative stress in the affective and cognitive disorders associated with PTX-induced chemotherapy has been previously demonstrated [23,51]; PTX-induced anxiety was found to be inhibited by compounds such as 7-chloro-4-(phenylselanyl) quinoline (4-PSQ) through the modulation of oxidative stress in specific brain areas [52]. Therefore, our results suggest that the antioxidant properties of MCC950 could also influence the control of the abnormal emotional behaviors induced by this drug.

As in the peripheral nervous system, the MCC950 treatment also inhibited the apoptotic responses provoked by PTX in the amygdala. These data contrast with the lack of changes in BAX expression in the prefrontal cortex of PTX-injected mice but are in agreement with the apoptotic effects provoked by this antineoplastic drug in other brain areas [53]. Our outcomes support the antiapoptotic effects induced by MCC950 in the sciatic nerve. Moreover, the fact that PTX increased NLRP3 expression in the amygdala and that the pharmacological inhibition of NLRP3 attenuated BAX overexpression and reversed the anxiety- and depressive-like behaviors associated with PTX suggests that cell death might also be involved in the development of the emotional alterations provoked by this chemotherapeutic agent.

In this work, we also evaluated the influence of the NLRP3 inflammasome on the cognitive impairments provoked by PTX by assessing the effects of the MCC950 treatment on novel object recognition test performance. In accordance with the results of Liang et al. (2020) [54], our data revealed a decreased discrimination index in PTX-injected mice, demonstrating the memory loss induced by this antineoplastic drug. Moreover, this memory deficit was rescued with the MCC950 treatment, which indicates that the NLRP3 inflammasome is involved in the development of the cognitive deficits related to PTX chemotherapy. This is in accordance with other works that proved that central inflammation is one of the primary causes of the cognitive deficits related to chemotherapy [55,56]. Increased levels of the NLRP3 inflammasome were found in the hippocampus of PTX-injected animals, and the administration of MCC950 normalized the up-regulation of this inflammasome. Since the hippocampus is strongly implicated in memory processes [57], inhibition of NLRP3 up-regulation by MCC950 suggests that the attenuation of the memory deficits induced by this treatment was due to a reduction in inflammatory pathways activated by this inflammasome. This idea is supported by recent preclinical studies that showed that MCC950 rescued the memory deficits caused by orthopedic surgery through normalizing the increased interleukin levels in the hippocampus [58] and that molecular hydrogen attenuated the cognitive impairments caused by vascular dementia by inhibiting NLRP3 inflammasome activation in the hippocampus [59].

Several studies have demonstrated that chemotherapy causes oxidative stress in the hippocampus [60,61]. Our results support these data, since they demonstrated decreased expression of HO-1, NQO1, and SOD-1 in this brain area in mice given PTX. Unexpectedly, decreased levels of 4-HNE and BAX were also found in the hippocampi of these animals, both of which were normalized by the MCC950 treatment. We do not have a clear explanation for these results, but we hypothesize that they may be due to a possible compensatory mechanism that maintains hippocampal homeostasis when there is a decline in the activity of the endogenous antioxidant system. Nevertheless, a complete normalization of the HO-1, NQO1, and SOD-1 levels was observed in the animals treated with MCC950, demonstrating the antioxidant actions of this drug in the hippocampus, which may be responsible for the rescue of the memory deficits. This hypothesis is supported by the observed alleviation of memory loss by some antioxidants, such as vitamin E, in rats exposed to chemotherapy through a reduction in hippocampal oxidative stress [62].

Similar to results in the prefrontal cortex [22,23], elevated levels of p-ERK 1/2 were observed in the hippocampus of PTX-injected mice that were not reversed by the treatment with MCC950. Therefore, the mechanism through which MCC950 improves memory loss does not seem to be related to the p-ERK 1/2 signaling pathway, which was also observed with other treatments [22].

Finally, our data showed that the acute administration of HRW enhanced the mechanical and thermal antiallodynic effects of a single dose of MCC950, demonstrating an additive effect of the NLRP3 inflammasome and molecular hydrogen administration in modulating the allodynia caused by PTX. This interaction is further reinforced by the normalization of the up-regulation of NLRP3 induced by HRW in the dorsal root ganglia of animals with PTX-induced neuropathy [35]. In agreement with these findings, a positive interaction between molecular hydrogen and the antioxidant enzyme HO-1 was also found to alleviate the allodynia caused by nerve injury [33]. Therefore, considering the limited side effects produced by the HRW [63], our results suggest that the combination of MCC950 and HRW could be a promising and safer approach for addressing the neuropathy induced by PTX.

One limitation of this study is that the experiments were only performed on male mice. However, considering the positive effects of MCC950 on the male mice, we plan to perform these experiments on female mice in the near future.

## 5. Conclusions

In summary, this investigation revealed that the administration of MCC950 attenuated the allodynia produced by PTX, and the strength of this effect was increased by co-administration with HRW. The data further demonstrated the anxiolytic and antidepressant effects of MCC950 and its ability to rescue memory deficits in PTX-injected mice. These actions were associated with lower NLRP3 inflammasome activation in the sciatic nerve, amygdala, and hippocampus and less oxidative stress in the amygdala and hippocampus. MCC950 also inhibited p-ERK 1/2 overexpression in the sciatic nerve and reduced the apoptotic responses provoked by PTX in the sciatic nerve and amygdala. In conclusion, this study reveals that MCC950 represents a promising therapeutic option for ameliorating neuropathic pain and its associated mood and cognitive disorders.

## Figures and Tables

**Table 1:** Primary antibodies.

Antibody	Dilution	Commercial Source
NLRP3	1:200	Adipogen Life Sciences, Epalinges, Switzerland
4-HNE	1:150	Abcam, Cambridge, United Kingdom
p-ERK 1/2	1:250	Cell Signaling Technology, Danvers, MA, USA
ERK 1/2	1:250	Cell Signaling Technology, Danvers, MA, USA
HO-1	1:150	Enzo Life Sciences, New York, NY, USA
NQO1	1:250	Merck, Billerica, MA, USA
SOD-1	1:150	Novus Biologic, Littleton, CO, USA
BAX	1:150	Cell Signaling Technology, Danvers, MA, USA
GAPDH	1:5000	Merck, Billerica, MA, USA

## Data Availability

The data presented in this study are available in the article.
